# Supplementary material for: Intelligent classification of platelet aggregates by agonist type
Source: eLife. 2020 May 12;9:e52938. doi: 10.7554/eLife.52938 (PMC7217700; doi:10.7554/eLife.52938)
Supplement: Transparent reporting form [file elife-52938-transrepform.docx]

***eLife’s* transparent reporting form**

We encourage authors to provide detailed information *within their submission* to facilitate the interpretation and replication of experiments. Authors can upload supporting documentation to indicate the use of appropriate reporting guidelines for health-related research (see [EQUATOR Network](http://www.equator-network.org/%20)), life science research (see the [BioSharing Information Resource](https://biosharing.org/)), or the [ARRIVE guidelines](http://www.plosbiology.org/article/info:doi/10.1371/journal.pbio.1000412) for reporting work involving animal research. Where applicable, authors should refer to any relevant reporting standards documents in this form.

If you have any questions, please consult our Journal Policies and/or contact us: [editorial@elifesciences.org](mailto:editorial@elifesciences.org).

**Sample-size estimation**

- You should state whether an appropriate sample size was computed when the study was being designed
- You should state the statistical method of sample size computation and any required assumptions
- If no explicit power analysis was used, you should describe how you decided what sample (replicate) size (number) to use

Please outline where this information can be found within the submission (e.g., sections or figure legends), or explain why this information doesn’t apply to your submission:

For image classification using deep learning, the empirical bound for training data size is 1,000 images per class at least (Krizhevsky et al. *Communications of the ACM.* 60 (6): 84–90). As stated in the section titled ‘Development of the iPAC’, we used 12,000 images per class in our work. This data size is much larger than the empirical size so that the bias from the fluctuation of image quality can be minimized in the classification process.

**Replicates**

- You should report how often each experiment was performed
- You should include a definition of biological versus technical replication
- The data obtained should be provided and sufficient information should be provided to indicate the number of independent biological and/or technical replicates
- If you encountered any outliers, you should describe how these were handled
- Criteria for exclusion/inclusion of data should be clearly stated
- High-throughput sequence data should be uploaded before submission, with a private link for reviewers provided (these are available from both GEO and ArrayExpress)

Please outline where this information can be found within the submission (e.g., sections or figure legends), or explain why this information doesn’t apply to your submission:

The experiment was performed as six replications for developing the classification model. As stated in the ‘Development of the iPAC’ section, multiple experimental replications were performed in order to mitigate potential bias in the dataset that may have come from experimental variations (e.g., signal-to-noise ratio, fluctuations in optical alignment, hydrodynamic cell focusing conditions, sample preparation). Although the fluctuations in image quality existed, no outlier was encountered in all the replications. Blurring images (less than 10%) were excluded in image-based classification.

**Statistical reporting**

- Statistical analysis methods should be described and justified
- Raw data should be presented in figures whenever informative to do so (typically when N per group is less than 10)
- For each experiment, you should identify the statistical tests used, exact values of N, definitions of center, methods of multiple test correction, and dispersion and precision measures (e.g., mean, median, SD, SEM, confidence intervals; and, for the major substantive results, a measure of effect size (e.g., Pearson's r, Cohen's d)
- Report exact p-values wherever possible alongside the summary statistics and 95% confidence intervals. These should be reported for all key questions and not only when the p-value is less than 0.05.

Please outline where this information can be found within the submission (e.g., sections or figure legends), or explain why this information doesn’t apply to your submission:

The images were analyzed and classified using deep learning. Figure 3B shows the mapping of data distribution in the high-dimensional space into the 2-dimensional space. The details of the convolutional neural network are described in Materials and Methods. The dispersion and precision measures for statistical tests are not applicable to the deep-learning-based analysis in this work.

(For large datasets, or papers with a very large number of statistical tests, you may upload a single table file with tests, Ns, etc., with reference to sections in the manuscript.)

**Group allocation**

- Indicate how samples were allocated into experimental groups (in the case of clinical studies, please specify allocation to treatment method); if randomization was used, please also state if restricted randomization was applied
- Indicate if masking was used during group allocation, data collection and/or data analysis

Please outline where this information can be found within the submission (e.g., sections or figure legends), or explain why this information doesn’t apply to your submission:

The images of platelet aggregates activated by the same agonist were allocated as one group. Also, the images of platelets without any activation by agonists were used as a negative control group. No randomization or masking was used in this work.

**Additional data files (“source data”)**

- We encourage you to upload relevant additional data files, such as numerical data that are represented as a graph in a figure, or as a summary table
- Where provided, these should be in the most useful format, and they can be uploaded as “Source data” files linked to a main figure or table
- Include model definition files including the full list of parameters used
- Include code used for data analysis (e.g., R, MatLab)
- Avoid stating that data files are “available upon request”

Please indicate the figures or tables for which source data files have been provided:

The table of the details in the analysis of agonist-activated platelets by conventional flow cytometry is shown in the Supplementary Information as Figure2-source data1. The programs for image recovery in Matlab and image classification in Python are available at <https://github.com/irisaya/IPAC>.
